# Characterization of major metabolites of polymethoxylated flavonoids in Pericarpium Citri Reticulatae using liver microsomes immobilized on magnetic nanoparticles coupled with UPLC/MS–MS

**DOI:** 10.1186/s13065-017-0237-9

**Published:** 2017-02-06

**Authors:** Jun Lei, Ying Xue, Yi-Ming Liu, Xun Liao

**Affiliations:** 1 Institute of Chemistry and Chemical Engineering, Mianyang Normal University, Mianyang, 621000 China; 2 Sichuan Centre for Disease Control and Prevention, Chengdu, 610041 China; 3 Chengdu Institute of Biology, Chinese Academy of Sciences, Chengdu, 610041 Sichuan China

**Keywords:** Liver microsome, Magnetic nanoparticles, Metabolism, Polymethoxylated flavonoids, Pericarpium Citri Reticulatae

## Abstract

The peels of citrus fruits (Pericarpium Citri Reticulatae, PCR) have long been used in traditional Chinese medicines (TCMs). Polymethoxylated flavonoids (PMFs) were found to be the main components present in PCR extracts, but their metabolism remains unclear which restrain the utilization of this TCM. In the present work, rat liver microsomes were immobilized on magnetic nanoparticles (LMMNPs) for in vitro metabolic study on the whole PMFs of PCR. LMMNPs were characterized by transmission electron microscope and Fourier-transform infrared spectrum. The relative enzyme binding capacity of LMMNPs was estimated to be about 428 μg/mg from thermogravimetric analysis. Incubation of LMMNPs with PMFs produced demethylated metabolites of PMFs, six of which were identified by ultrahigh pressure liquid chromatography–mass spectrometry (UPLC–MS/MS). The 3′-hydroxylated tangeretin (T3) was detected from the metabolites of tangeretin for the first time, which suggested that 4′-demethylated and 3′-hydroxylated derivative of tangeretin (3′-hydroxy-5,6,7,8,4′-pentamethoxyflavone, T4) was not only derived from 4′-demethylated tangeretin (T2) as previously reported, but also from T3. This is the first investigation of the metabolism of the whole PMFs, which may shed light on the mechanism of action of PCR.

## Background

The health benefits and economic values of peels from citrus fruits have been known for over a thousand years, and they have now been widely used in pharmaceutical, food and cosmetic industries. In traditional Chinese medicine, the dried ripe pericarps of *Citrus reticulata* or its cultivars, namely Pericarpium Citri Reticulatae (PCR, Chenpi in Chinese) were used to treat chronic diseases, such as coughing, stomach upset, and skin inflammation with great medicinal values [1]. Polymethoxylated flavonoids (PMFs) were the major components in PCR [2–5], and increasing evidence shows that PMFs possess several protective effects including anti-oxidant, anti-inflammation, anti-proliferation, anticancer, cardiovascular protection and so on [6–9]. The planar structures and the low polarity of PMFs might enhance their permeability to biological membranes, thus endow the PMFs with high bioavailability [10, 11]. Therefore, PMFs have attracted increasingly attention in the development of specialty ingredients for nutraceutical and pharmaceutical industries.

The metabolic study on the active components present in the herbal extracts is important for understanding the mechanism of action for the original TCMs. Biotransformation of two PMFs isolated from PCR, nobiletin and tangeretin, has been investigated both in vitro and in vivo. In vitro experiments showed that demethylated derivatives were their major metabolites [12–14]. In vivo study in the mouse revealed that 4′-demethylnobiletin was the major metabolite of nobiletin [15], while demethylated and hydroxylated products are the major metabolites for tangeretin [16]. Despite all those studies on individual PMFs, there have no reports on the metabolism of the whole extract of the PCR. Since PCR has been utilized in the form of mixture like most herbals do, investigation of the metabolism of its whole extracts can provide more reasonable information in understanding the mechanism of action.

In this study, we investigated the metabolism of the whole extracts of PCR by a highly active nanobioreactor prepared by immobilizing rat liver microsomes onto magnetic nanoparticles (LMMNPs). Magnetic nanoparticles have been widely used as adsorbent to immobilize biomolecules for the enrichment of natural products due to the convenience of magnetic solid–liquid separation [17–19]. Previously, we have developed a similar microsomal nanobioreactor for the in vitro metabolic study of *Rhizoma coptidis* extract which exhibited higher activity and stability than free microsomes [20]. In the present work, we improved the relative enzyme loading capacity of the microsomal nanobioreactors that greatly facilitated metabolic study on the whole extract of PCR. The metabolites of PCR extract were characterized by ultra-high pressure liquid chromatography–mass spectrometry (UPLC–MS/MS), most of which were compared with those of nobiletin and tangeretin which are the two major PMFs present in PCR extract.

## Experimental

### Materials and reagents

Pericarpium Citri Reticulatae was purchased from a local herbal market. It was authenticated by Professor Xin-feng Gao. A voucher specimen was deposited in Herbaruim of Chengdu Institute of Biology, No. CIBI0064257.

Nobiletin and Tangeretin were purchased from Chengdu Must Bio-technology Co., LTD (China) and indentified in our laboratory for qualitative and quantitative analysis. β-naphthoflavone, polydiallyldimethylammonium chloride (PDDA), β-nicotinamide adenine dinucleotide phosphate hydrate (NADP), glucose-6-phosphate, yeast glucose-6-phosphate dehydrogenase, 4-nitrophenol (PNP) and 4-nitrocatechol (PNC) were purchased from Sigma (MO, USA). HPLC grade acetonitrile was purchased from Fisher Scientific (Fisher, Fair Lawn, USA). Deionized water was purified by a Milli-Q water system (Millipore Corp., Bedford, MA, USA). Tetraethyl orthosilicate (TEOS) was purchased from TCI (Tokyo, Japan). Other chemicals and solvents were of analytical reagent grade and were obtained from Chengdu Chemical Factory (Chengdu, China).

### Sample preparation

Pericarpium Citri Reticulatae was dried and powdered, and 1 g of the sample was placed into a 250 mL conical flask containing 100 mL methanol to be refluxed in water bath at 90 °C for 1 h. The methanol solution was filtered and cooled to the room temperature before used. Nobiletin and tangeretin were respectively dissolved in methanol at 1 mg/mL as work solutions.

### Rats liver microsomes preparation

Microsomes were prepared from the livers of β-naphthoflavone treated male Sprague–Dawley rats according to standard procedures described by Lake [21]. The inductor was dissolved in vegetable oil at 8 mg/mL, and was intraperitional injected to the rats at a dose of 80 mg/kg once for two days. The rats were sacrificed in the third day for the microsome preparation. The protein concentrations of the microsome obtained were estimated by Bradford assay using the bovine serum albumin (BSA) as standard [22].

### Nanobioreactor fabrication

The microsomes were immobilized onto the MNPs according to the following procedure. MNPs were synthesized by co-precipitation and coated with a layer of SiO_2_ using TEOS, and were then dispersed in 2 mg/mL PDDA for 20 min to distribute a layer of positive change on the surface. The PDDA-MNPs were dispersed in microsomes dispersion for 30 min to absorb the liver microsomes. Finally, an external magnet was used to separate the resultant magnetic nanoparticles from the solution to obtain the final bioreactor (LMMNPs). The sizes and morphologies of magnetic nanoparticles were recorded using a transmission electron microscope (TEM, H-600IV, Hitachi Co., Tokyo, Japan). The Fourier-transform infrared spectra (FT-IR) were obtained with a Perkin-Elmer Spectrum 100 (Waltham, MA, USA). Thermogravimetric analysis (TGA) was performed for powdered samples with a heating rate of 10 °C/min^1^ from room temperature to 800 °C under nitrogen atmosphere using a TGA Q500 V20.10 Build 36 thermo analysis system (TA instruments, New Castle, USA).

### Metabolism kinetics

The PNP was used as the substrate to compare the enzymatic activity of the LMMNPs with the free microsomes. When PNP is incubated with the microsomes, the microsomes mediate its hydroxylation to produce PNC. This reaction has usually been used to test hepatic activity in different animal species. In this experiment, PNP was incubated with LMMNPs and free microsomes respectively to compare the Michaelis constant (*K*_m_) and the maximum rate of the reaction (*V*_max_), during which the amount of microsmes immobilized in LMMNPs was equivalent to that of the free microsomes.

### Incubation

Metabolisms of nobiletin, tangeretin and the extract of Pericarpium Citri Reticulatae were studied by incubating 10 μL work solution of each with 100 μL of bioreactor dispersion in 0.1 M potassium phosphate buffer (pH 7.4), and the incubation volume was finally adjusted to 400 μL containing 10 mM magnesium chloride. The metabolic reaction was initiated by adding 100 μL of NADPH-generating solution (1.3 mM NADP, 3.3 mM glucose-6-phosphate and 1 U/mL yeast glucose-6-phosphate dehydrogenase) and incubated at 37 °C for 60 min. The reaction was terminated by magnetic separation of the bioreactors from the reaction solution, and the supernatant was filtered though a 0.22 μm membrane and subjected to UPLC–MS/MS analysis. No organic solvent were added to stop the reaction as do in other enzymatic reactions, so that the LMMNPs separated can be reused after washing with potassium phosphate buffer(0.1 M, pH 7.4) three times (500 μL each time).

### Instrumentation

The Waters ACQUITY ultra-performance liquid chromatographic systems (Waters, Milford, PA, USA) used in this experiment was equipped with a binary pump, an autosampler, a photodiode array detector, a column temperature controller and a Waters xevo™ mass Spectrometer with triple-quadrupole MS system. The analytes were separated on a Waters ACQUITY UPLC BEH C18 column (2.1 × 100 mm, 1.7 μm). Formic acid aqueous solution (0.1% formic acid, solvent A) and acetonitrile (solvent B) were used as mobile phase for UPLC separation. The elution condition was as follows: 20% B at 0–1 min, 20–40% B at 1–3 min, 40–95% B at 3–7 min. The wavelength of PDA detector was set in the range of 200–400 nm. The flow rate was set at 0.2 mL/min and the peaks were detected at 345 nm. Moreover, the autosampler temperature was kept at 10 °C, and a 1 μL of each sample was injected for analysis. ESI–MS spectra were acquired in positive ion mode in the range of *m*/*z* 100–1000 for the full-scan MS analysis. The source parameters were set as follows: the capillary voltage was 3.25 kV, the cone voltage was 50 V, the source and desolvation temperatures were set at 100 and 350 °C. Nitrogen was used as the desolvation gas at a flow rate of 550 L/h, and argon was used as collision gas at a flow rate of 0.15 mL/min.

## Results and discussion

### Synthesis and characterization of LMMNP nanobioreactors

**Fig. 1:**
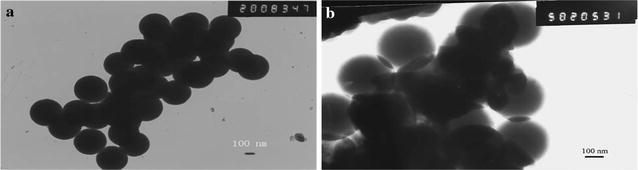
TEM images of **a** Fe_3_O_4_@SiO_2_ magnetic nanoparticles and **b** LMMNP nanoparticle bioreactors

**Fig. 2:**
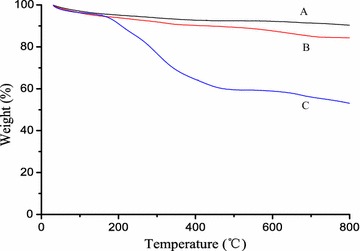
TGA analysis of (*A*) Fe_3_O_4_@SiO_2_, (*B*) Fe_3_O_4_@SiO_2_@PDDA and (*C*) LMMNPs

Electrostatic adsorption method was applied to prepare the superparamagnetic nanocomposites. The size of the magnetic nanoparticles was measured by TEM. Figure 1a shows that the average diameter of the Fe_3_O_4_@SiO_2_ was about 200 nm, and small protuberances can be seen at the edges of the nanoparticles of LMMNPs in Fig. 1b. Moreover, the LMMNP nanoparticle bioreactors accumulated rapidly in solution under magnetic field and dispersed quickly with a slight shake once the magnetic field was removed, which indicated that the LMMNP nano-bioreactors were successfully synthesized. FT-IR spectra provide further evidence for the successful immobilization of liver microsomes onto the surface of magnetic nanoparticles. The peak at 1084 cm^−1^ is assigned to the silica layer vibrations. The band between 3300 and 3400 cm^−1^ results from the stretching vibration of the hydroxyl group. Compared with Fe_3_O_4_@SiO_2_ and Fe_3_O_4_@SiO_2_@PDDA, the absorption peaks at 2924, 2853, 1647, 1542 and 1457 cm^−1^ for LMMNPs are ascribable to the vibrations of the methylene and amide groups, which belong to side chains of the enzyme on the surface of the magnetic nanoparticles [23, 24]. The relative enzyme loading capacity was estimated to be about 428 μg protein/mg MNPs based on the results from the TGA analysis (Fig. 2).

### Kinetic constants

Kinetic parameters, the Michaelis constant (*K*_m_) and the maximum rate of the reaction (*V*_max_) for free and immobilized rat liver microsomes were assayed using the PNP as substrate. *K*_m_ and *V*_max_ were calculated from the Lineweaver–Bruk plots using the initial rate of the reaction data.

$$ V = \frac{{V_{\hbox{max} } \times [S]}}{{K_{\text{m}} + [S]}} $$

$$ \frac{1}{V} = \frac{{K_{\text{m}} }}{{V_{\hbox{max} } }} \times \frac{1}{[S]} + \frac{1}{{V_{\hbox{max} } }} $$

where [*S*] is the concentration of the substrate, *V* and *V*_max_ represent the initial and the maximal rate of the reaction, respectively, and *K*_m_ is the Michaelis constant. *V*_max_ is defined as the highest possible velocity when all enzymes are saturated with the substrate, reflecting the intrinsic characteristics of the immobilized enzymes. *K*_m_ is defined as the substrate concentration that yields a reaction velocity of 1/2, reflecting the effective characteristics of the enzyme. The values of *K*_m_ and *V*_max_ were calculated from Lineweaver–Burk plots as shown in Table 1. The *K*_m_ for LMMNPs is in the same order with that of free microsomes, indicating that the enzymatic activity of LMMNPs was acceptable for the following metabolic study.

**Table 1:** *K*_m_ and *V*_max_ for free and immoblized rat liver microsomes for PNP

Form of microsomes	*K*_m_ (mM)	*V*_max_ (nM/min/mg)
Free microsomes	0.15	200
LMMNPs	0.45	

In the mean time, the prepared LMMNPs exhibited good reusability as we described in our previous paper, in that it retained its original enzymatic activity after six rounds of use [20]. Owing to the superparamagnetism of the LMMNPs, it is very convenient to stop the enzymatic reaction and separate the bioreactor from the incubation solution by using an external magnet, while at the same time, the supernatant can be directly subjected for HPLC–MS for analysis of the metabolites.

### The metabolite profiling of the extract of Pericarpium Citri Reticulatae

**Fig. 3:**
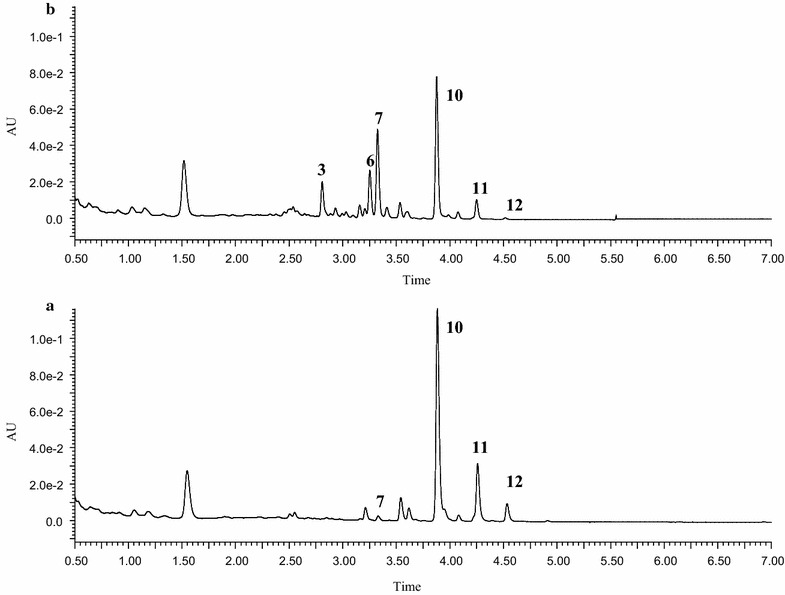
The UPLC analysis of the whole extract of PCR incubation solution **a** 0 min; and **b** 60 mmin. Compounds represented by peaks 3–12 are listed in Table 2

Polymethoxylated flavonoids such as nobiletin and tangeretin were reported to be the major active components in PCR, and there have been intensive in vitro studies on the biotransformation of individual polymethoxylated flavonoids. However, to the best of our knowledge, no in vitro metabolic study on the whole extract of PCR has been performed. As there are multiple active components in herbs, in vitro metabolism of individual compounds might not reflect the real fate of the herbs. In this work, we incubated the PCR extract with LMMNPs in order to investigate the metabolites of the whole extract. The UPLC–MS/MS chromatograms obtained from the incubation solution for 0 and 60 min are shown in Fig. 3. It was found that the peak areas of peaks 10, 11 and 12 were significantly reduced after metabolization. These compounds were identified as nobiletin, tangeretin and monohydroxy pentamethoxyflavone according to the retention times, UV absorption spectra and MS/MS fragments. In addition, several new peaks were observed between 2.50 and 3.50 min in the 60 min incubation solution. Based on the UV absorption spectra, the molecular ions [M+H]^+^ and the MS/MS fragment ions, these compounds were tentatively identified as dihydroxytetramethoxyflavone ([M+H]^+^ *m*/*z* 375), monohydroxypentamethoxyflavone ([M+H]^+^ *m*/*z* 389) and monohydroxytetramethoxyflavone ([M+H]^+^ *m*/*z* 359), respectively [2].

**Fig. 4:**
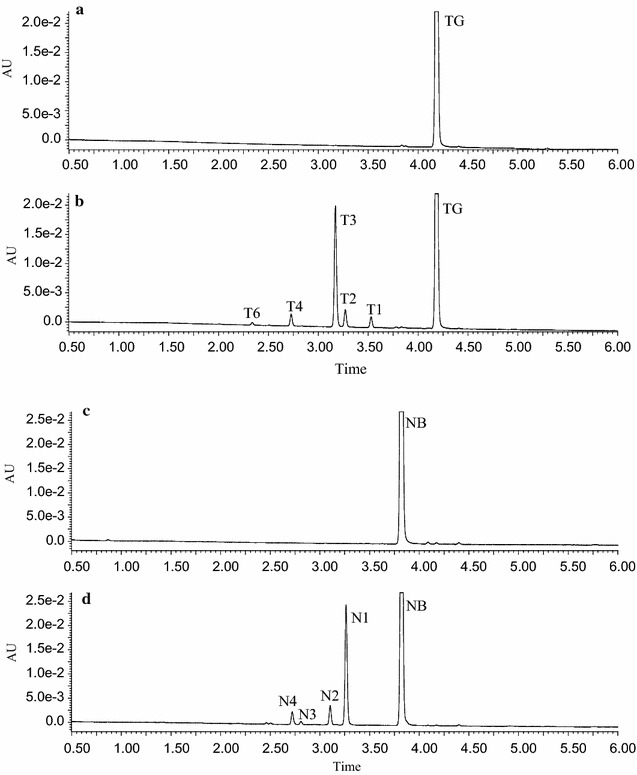
The UPLC chromatograms of tangeretin and nobiletin solutions incubated with LMMNPs: **a** tangeretin + LMMNPs at 0 min; **b** tangeretin + LMMNPs at 60 min; **c** nobiletin + LMMNPs at 0 min; and **d** nobiletin + LMMNPs at 60 min

**Fig. 5:**
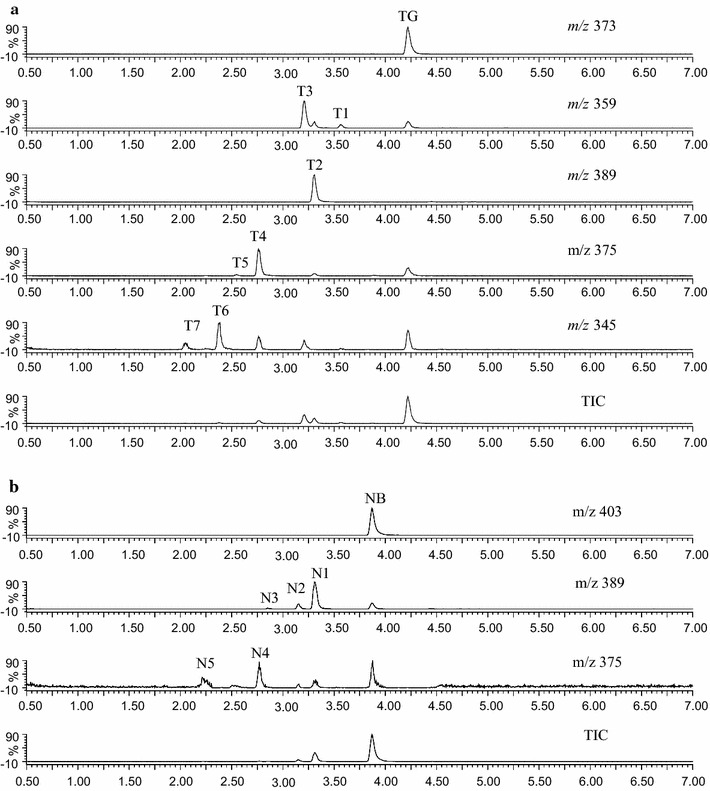
UPLC–MS chromatograms from the incubation solution at 60 min. **a** Tangeretin; **b** nobiletin

**Fig. 6:**
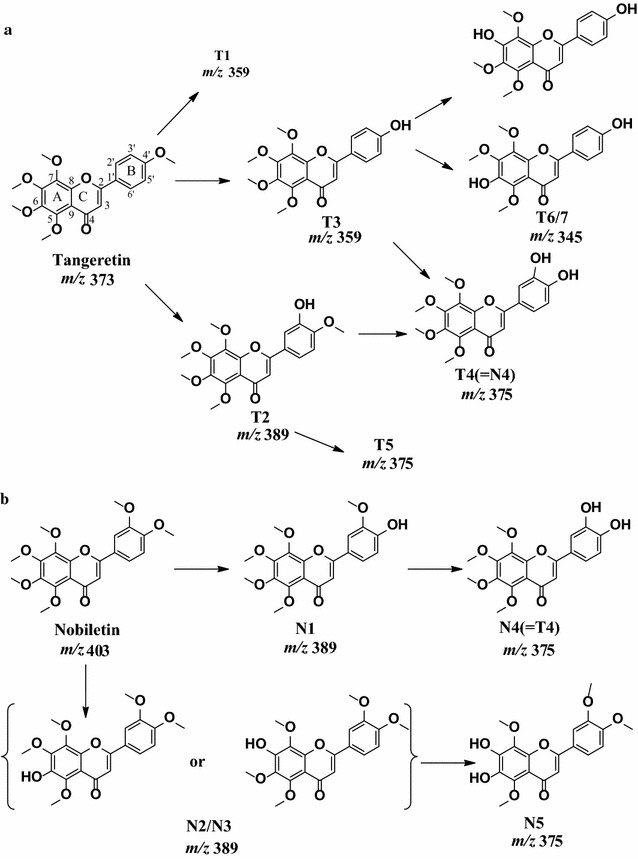
The proposed metabolic pathways of tangeretin (**a**) and nobiletin (**b**)

To further verify the structures of the above metabolites, the two major components of the PCR, tangeretin and nobiletin were metabolized respectively with LMMNPs. The UPLC–UV–MS/MS indicated that seven metabolites were formed from tangeretin as shown in Figs. 4a, b and 5a. The three major peaks of T2, T3 and T4, with molecular masses of 388 ([M+H]^+^ *m*/*z* 389), 358 ([M+H]^+^ *m*/*z* 359) and 374 ([M+H]^+^ *m*/*z* 375), respectively, were tentatively identified as 3′-hydroxy-5,6,7,8,4′-pentamethoxyflavone, 4′-hydroxy-5,6,7,8-tetramethoxyflavone and 3′,4′-dihydroxy-5,6,7,8-tetramethoxy flavone, according to a previous report on the metabolism of tangeretin [25]. In this reports, tangeretin was metabolized firstly to 4′-demethylated tangeretin (4′-hydroxy-5,6,7,8-tetramethoxyflavone, T3), and then a hydroxyl group was added to the C-3′ of T3 to form 3′,4′-dihydroxy-5,6,7,8-tetramethoxy flavone (T4). Interestingly, 3′-hydroxylated tangeretin (T2) was detected as the metabolite of tangeretin for the first time, suggesting a new metabolic pathway in which T4 might also be transferred via T2. In addition, T1 and T5 had different retention time but the same molecular mass with T3 ([M+H]^+^ *m*/*z* 359) and T4 ([M+H]^+^ *m*/*z* 375), respectively, which indicates that their structures were similar to T3 and T4. The other two metabolites (T6 and T7) had the same molecular mass of 344 ([M+H]^+^ *m*/*z* 345) but different retention time, suggesting the loss of one methyl group at various positions of T3, and they were tentatively identified as 4′,6-dihydroxy-5,7,8-trimethoxyflavone or 4′,7-dihydroxy-5,6,8-trimethoxyflavone. The proposed metabolic pathway of tangeretin was shown in Fig. 6a.

Five metabolites were detected for the nobiletin in UPLC–UV–MS/MS as shown in Figs. 4c, d and 5b. The molecular masses of the metabolites are *m*/*z* 389 (N1, N2 and N3), *m*/*z* 375 (N4 and N5), respectively. The major metabolite N1 was tentatively identified as 4′-hydroxy-5,6,7,8,3′-pentamethoxyflavone, which was a 4′-demethylated product of nobiletin. N2 and N3 were tentatively detected as 6-hydroxy- 5,7,8,3′,4′-pentamethoxyflavone or 7-hydroxy-5,6,8,3′,4′-pentamethoxyflavone, which were formed by the loss of one methyl group at 6 or 7 position in nobiletin. N4 and N5 were found to be the demethylated products of N1 and N2 (or N3), respectively, which were tentatively identified as 3′,4′-dihydroxy-5,6,7,8-tetramethoxyflavone (N4) or 6,7-dihydroxy-5,8,3′,4′-tetramethoxyflavone (N5), as show in Fig. 6b. These results are in agreement with a previous report on the metabolism of nobiletin catalyzed by rat liver microsomes [12]. It is worth noting that N4 was identical to T4 based on the retention time, molecular mass and UV absorption.

From the above description, it is obvious that the major metabolites of PCR extract were the same with those of nobiletin and tangeretin. However, in the HPLC chromatogram of the PCR extract metabolites, complicated cases happened in which different parent compounds produced the same metabolite. For example, T4 was identical to N4 (Fig. 6), while this metabolite can be derived from both tangeretin and nobiletin. On the other hand, a metabolite could be identical to a compound originally present in the extract, as is the case of peak 7 (Fig. 3). The metabolite profile of the extract of PCR is summarized in Table 2.

**Table 2:** The compounds identified in incubation solutions at 0 min (M0) and 60 min (M1), respectively

Peaks	*t*_R_ (min)	M0 [M+H]^+^	M1 [M+H]^+^	MS^2^	Identification
1	2.59	–	375	345	Di hydroxy tetramethoxyflavone
2	2.63	–	375	345	Di hydroxy tetramethoxyflavone
3	2.81	–	375	345, 331, 301	3′,4′-di hydroxy-5,6,7,8- tetramethoxy-flavone
4	3.16	–	389	359, 374, 341	6-hydroxy-5,7,8,3′,4′-pentamethoxy-flavone or 7-hydroxy-5,6,8,3′,4′-pentame-thoxyflavone
6	3.25	–	359	329	4′-hydroxy-5,6,7,8- tetramethoxyflavone
7	3.33	389	389	359, 374, 341	4′-hydroxy - 5,6,7,8,3′-pentamethoxyfla-vone or 3′-hydroxy- 5,6,7,8,4′-pentame-thoxyflavone
8	3.53	373	373	343, 312	Pentamethoxyflavone
9	3.60	359	359	329	Monohydroxy-tetramethoxyflavone
10	3.88	403	403	373	Nobiletin
11	4.25	373	373	343, 325	Tangeritin
12	4.53	389	389	359, 341, 374	Monohydryoxy- pentamethoxyflavone

## Conclusion

Liver microsomes immobilized on magnetic nanoparticles are proven to be reusable and very effective for in vitro metabolic studies. Thanks to the superparamagnetic property of these bioreactors, isolation of enzymes from the metabolic solution can be easily achieved by using an external magnet, avoiding tedious sample pretreatment such as centrifugation, filtration and evaporation which are normally required for metabolite analysis. Using the proposed nanobioreactors, in vitro metabolism of the whole extract of Pericarpium Citri Reticulatae was investigated in this work for the first time. Polymethoxylated flavonoids present in the extract and their metabolites were identified by UPLC–UV–MS/MS. Three polymethoxylated flavonoids in the PCR whole extract, i.e. nobiletin, tangeretin and monohydroxy pentamethoxyflavone were effectively metabolized by the LMMNPs bioreactors. Six metabolites, i.e. 3′,4′-dihydroxy-5,6,7,8-tetramethoxyflavone,4′-hydroxy-5,6,7,8-tetramethoxyflavone, 4′-hydroxy-5,6,7,8,3′-pentamethoxyflavone, 3′-hydroxy-5,6,7,8,4′-pentamethoxyflavone, 6-hydroxy-5,7,8,3′,4′-pentamethoxyfla- vone/7-hydroxy-5,6,8,3′,4′-pentamethoxyflavone and dihydroxy tetramethoxyflavone were tentatively identified from the metabolites mixture. Among them, 3′-hydroxy- 5,6,7,8,4′-pentamethoxyflavone was identified as the metabolite of a polymethoxylated flavonoid for the first time. This finding provides the first evidence that microsomal metabolism of polymethoxyflavones produce not only 4′-demethylated as documented in previous literatures, but also 3′-hydroxylated metabolites.

## Abbreviations

PCR: Pericarpium Citri Reticulatae
PMFs: polymethoxylated flavonoids
TCM: traditonal Chinese Medicine
LMMNPs: rat liver microsomes were immobilized on magnetic nanoparticles
UPLC–MS/MS: ultrahigh pressure liquid chromatography–mass spectrometry
PDDA: polydiallyldimethylammonium chloride
NADP: β-nicotinamide adenine dinucleotide phosphate hydrate
PNP: 4-nitrophenol
PNC: 4-nitrocatechol
TEOS: tetraethyl orthosilicate
TEM: transmission electron microscope
FT-IR: Fourier-transform infrared spectra
TGA: thermogravimetric analysis
